# Effects of lactic acid bacteria and cellulase additives on the fermentation quality, antioxidant activity, and metabolic profile of oat silage

**DOI:** 10.1186/s40643-024-00806-z

**Published:** 2024-10-01

**Authors:** Xin Wang, Han Liu, Yuan Wang, Yanli Lin, Kuikui Ni, Fuyu Yang

**Affiliations:** 1 College of Grassland Science and Technology, China Agricultural University, Beijing, 100193 China; 2 College of Animal Science, Guizhou University, Guiyang, 550025 China

**Keywords:** Oat silage, Additives, Antioxidant activity, Metabolite

## Abstract

Oats (*Avena sativa* L.) are rich in nutrients and bioactive compounds, serving as a roughage source for ruminants. This study investigated the impact of lactic acid bacteria (LAB), cellulase (M), and their combinations (LM) on the fermentation quality and metabolic compounds of oat silage. Results demonstrated that all additive treatments significantly increased lactic acid content compared to the control group (*P* < 0.05), with the lactic acid bacteria treatment group exhibiting the lowest pH value (*P* < 0.05). Analysis of antioxidant activity and metabolites in oat silage over 60 days revealed 374 differential metabolites with 113 up-regulated and 261 down-regulated, and all treatment groups showing higher antioxidant activity than raw oat materials (*P* < 0.05). Although no significant differences in antioxidant activity were observed among the various treatment groups in this experiment, notable changes in metabolic pathways were identified. Furthermore, two metabolites (carboxylic acids and derivatives and benzene and substituted derivatives) were identified through non-targeted metabolomics technology, both of which are strongly associated with the antioxidant activity of oat silage. This finding provides a theoretical basis for the efficient use of oat silage in animal husbandry.

**Graphical abstract:**

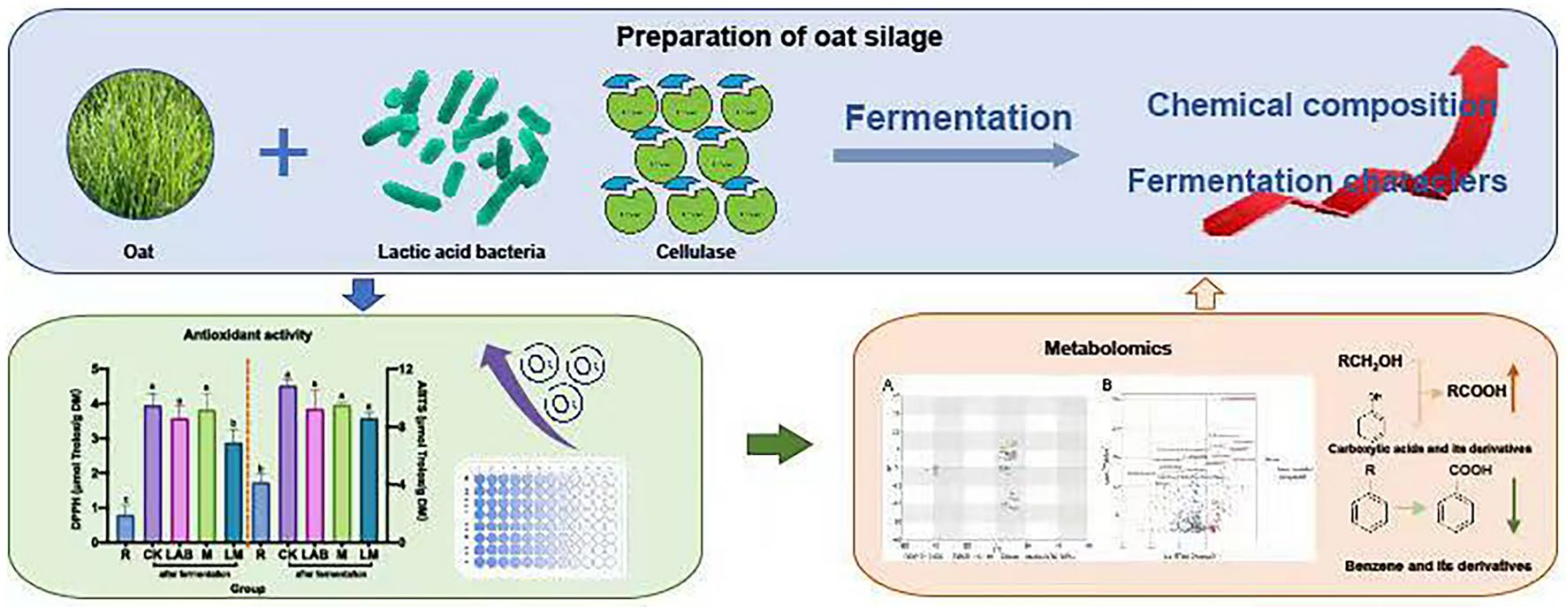

**Supplementary Information:**

The online version contains supplementary material available at 10.1186/s40643-024-00806-z.

## Introduction

Oats (*Avena sativa* L.), known for their high digestibility, palatability, and adaptability (Suttie and Reynolds 2004), have emerged as a significant roughage source for ruminants (Jia et al. 2021). Apart from their rich nutritional profile and feeding value, oats also boast various bioactive substances like beta-glucans, phenolic compounds, and alkaloids with antioxidant properties (Kim et al. 2021). Research indicates that flavonoids can effectively combat oxidative stress in animals, influencing intestinal mucosa morphology and the bacterial microecological environment (Ponnampalam et al. 2022). The modulation of feed antioxidant properties using terpenoids and alkaloids can boost livestock's antioxidant capacity and productivity, offering a novel approach to mitigate oxidative stress in animals (Tedeschi et al. 2021).

Ensiling plays a vital role in oat silage processing and storage. Research has shown that fermentation can greatly improve the quality and antioxidant properties of silage (He et al. 2020). Meeske (2002) demonstrated that using lactic acid bacteria composite inoculants can lower silage pH, increase dry matter content, and reduce butyric acid levels, thus ensuring better preservation quality. Additionally, incorporating cold-tolerant lactic acid bacteria into oat silage can enhance its quality and lower the final pH of the storage environment (Chen et al. 2020). Additives during ensiling fermentation can boost the antioxidant activity of silage feed by promoting biosynthesis and metabolism (Wang and Luo 2021; Zhang et al. 2022). For instance, the addition of cellulase has been found to increase the content of secondary metabolites like flavonoids and enhance antioxidant capacity (He et al. 2019). Bao (2016) noted a positive correlation between the antioxidant activity of mulberry leaves and total phenolic and flavonoid content. Similarly, He (2019) found that adding *Lactiplantbacillus plantarum* to mulberry silage can elevate flavonoid content and antioxidant activity. However, some studies suggest that inoculating *Lactiplantbacillus plantarum* and endo-1,4-β-glucanase during pumpkin (*Cucurbita maxima* D*.*) ensiling can lead to rapid oxidation of unstable fatty acids at lower pH values, along with increased total phenolic content, resulting in antioxidant properties similar to the raw material (Lozicki et al. 2015). Overall, research indicates that different additives used during the fermentation process can modulate various metabolic changes, resulting in diverse antioxidant outcomes in silage.

Limited research currently exists on the impact of various additives on controlling the antioxidant properties of oat silage. This study conducted experiments by introducing lactic acid bacteria (*Lactiplantbacillus plantarum* a214 and *Lactiplantbacillus brucei* 225) and cellulase, either individually or in combination, to analyze the alterations in oat silage quality, antioxidant activity, and metabolites throughout the fermentation process. The objective was to investigate the influence of different additives on the fermentation quality and antioxidant mechanism of oat silage.

## Materials and methods

### Experimental design and treatments

The oat silage material, specifically the Bayou No. 1 variety, was collected from Zhuozi County, Wulanchabu City, Inner Mongolia Autonomous Region (110°51' ~ 112°56' east longitude, 40°38' ~ 41°16' north latitude). The Oats were harvested at milky stage, and then ground into particles of 2–3 cm using a grinder. Distilled water, YiQing No. 3 (*Lactiplantbacillus plantarum* a214 + *Lactiplantbacillus brucei* 225, 1 × 10^6^ cfu /g), cellulase (50 mg/kg), YiQing No. 3 and cellulase were added separately to the mixture. After thorough mixing, the silage was vacuum sealed for fermentation on days 7, 14, 30, and 60, with each treatment having 4 replicates for a total of 128 bags. Subsequent analyses included silage fermentation characteristics, chemical composition, antioxidant activity evaluation, and non-targeted metabolome profiling.

### Silage preparation and sample collections

Oats weighing 500 g per treatment were placed into polyethylene standard barrier microporous bags (35 cm × 28 cm), which were then vacuumed and heat-sealed using a vacuum sealer. The samples were placed in a dark environment, fermented at room temperature (25–30 °C) and stored until opened.

### Analysis of fermentation characteristics

The oat silage samples were collected on the 7, 14, 30, and 60 d of ensiling. Each bag contained randomly weighed representative samples weighing 20 g of oat silage. Sterilized distilled water (180 mL) was added to each bag, Homogenization was carried out using a multifunctional temperature-controlled shaker (4 °C, 30 r/min, 4 h), and then filtered with 2 layers of sterile medical gauze. The filtrate was used for subsequent physical and chemical properties detection. The pH was measured using an electrode pH meter (PHS-3C, INESA Scientific Instrument, Shanghai, China). Organic acid contents including lactic acid, acetic acid, propionic acid, and butyric acid were determined using a high-performance liquid chromatograph (Showa Denko K.K., Kawasaki Japan) equipped with a DAD detector (210 nm) and a Shodex RS Pak KC-811 column after filtering the extract with a 0.22 μm aqueous filter. The mobile phase consisted of 3 mmol L^−1^ HClO_4_ flowing at a rate of 10 mL/min at a temperature of 50 °C. Ammonia nitrogen (NH_3_-N) levels were determined using Berthelot colorimetry (Broderick and Kang 1980).

### Analysis of chemical components

The oat silage samples were dried at 65℃ for over 48 h until a constant weight was achieved. The dry matter (DM) content was determined using weighing and counting methods. Total nitrogen content was analyzed using the Kjeldahl procedure (FOSS Kjeltec™ 2300) and crude protein (CP) was calculated by multiplying TN by 6.25 (Horwitz et al. 1980). Neutral detergent fiber (NDF) and acid detergent fiber (ADF) contents were measured following the method by Vansoest (Vansoest et al. 1991). Soluble carbohydrate (WSC) content was determined using anthrone-sulfuric acid colorimetry (Horwitz et al. 1980). Ether extract (EE) content was determined by extracting the fat using petroleum ether (Hasan 2015).

### Determination of antioxidant activity

Antioxidant activity was compared between raw oat material and oat silage fermented for 60 days. The samples were freeze-dried at -50℃ until a constant weight was achieved, followed by grinding using a laboratory grinder. Triplicate samples were prepared for subsequent analysis. The antioxidant capacity, including 2,2-diphenyl-1-picrylhydrazyl (DPPH) radical scavenging activity and 2,2-azinobis-3-ethylbenzothiazoline-6-sulfonic acid diammonium salt radical cation (ABTS) radical scavenging activity, was determined following the instructions provided in the kit from Suzhou Keming Biotechnology Co. LTD.

The DPPH radical scavenging activity assay was conducted as follows: 0.1 g of freeze-dried sample was weighed and combined with 1 mL of extract liquid (60% alcohol) by vigorous shaking at 160 r/min, 4℃ for 2 h. And then following centrifugation at 10,000 g and 4℃ for 10 min, the supernatant was collected and kept on ice for further analysis. Subsequently, 20 μL (diluted × 200) of the sample was added to a measuring tube, while distilled water was added to a blank tube, both followed by thorough mixing with 380 μL of reagent 1 (This reagent is sourced from the aforementioned kit, and the following is the same). The reaction mixture was then incubated at room temperature in the absence of light for 20 min. Absorbance values at a wavelength of 515 nm were determined using Varioskan LUX by transferring 200 μL of the reaction mixture into a well plate.

The ABTS radical scavenging activity assay was conducted by homogenizing a freeze-dried 0.1 g sample with 1 mL of extract liquid (60% alcohol) by vigorous shaking at 160 r/min, 4℃ for 2 h. After centrifugation at 10,000 g and 4℃ for 10 min, the supernatant was collected and kept on ice for measurement. A working liquid was prepared by mixing 11 mL of reagent 1 with reagent 2 for 20 min. In separate measuring and blank tubes, 10 μL of the test liquid and distilled water were added along with 190 μL of working liquid. The mixture was thoroughly mixed, and the absorbance value at 734 nm was measured within 10 min using Varioskan LUX.

### Determination of metabolites

The freeze-dried samples were rapidly frozen in liquid nitrogen for 30 min, followed by grinding for 1.5 min at 30 Hz using the freezing mixing ball mill (MM 400, Retsch) to obtain a powdered form. The entire powder was collected and homogenized. A freeze-dried sample weighing 50 mg was precisely measured and combined with 600 μL of methanol (containing 2-chloro-L-phenylalanine at a concentration of 4 ppm, stored at -20℃) in a 2 mL centrifuge tube centrifuge tube. The mixture was swirled for 30 s. Subsequently, glass beads weighing 100 mg were added to the tissue grinder and ground for 90 s at a frequency of 60 Hz. After ultrasonication for 15 min at room temperature and centrifugation at 12,000 rpm for 10 min at 4℃, the supernatant was filtered through a 0.22 μm filter membrane. The obtained filtrate was then added to the test bottle for detection using LC–MS (Vasilev et al. 2016).

Chromatography was performed using the Thermo Vanquish ultra-high performance liquid phase system (Thermo Fisher Scientific, USA) with an ACQUITY UPLC® HSS T3 column (2.1 × 150 mm, 1.8 µm) (Waters, Milford, MA, USA) at a flow rate of 0.25 mL/min and a column temperature of 40 ℃. A sample size of 2 μL was utilized. The mobile phase in positive ion mode comprised 0.1% acetonitrile format and 0.1% formic acid water. The gradient elution process involved specific steps: 0–1 min at 2% C, 1–9 min increasing from 2 to 50% C, 9–12 min increasing from 50 to 98% C, 12–13.5 min maintaining at 98% C, 13.5–14 min decreasing from 98 to 2% C, and finally maintaining at 2% C from 14 to 20 min. In the negative ion mode, the mobile phase consisted of acetonitrile (A) and a solution of ammonium formate in water with a concentration of five millimolars. The gradient elution procedure involved the following steps: starting at zero minutes with two percent A, gradually increasing from two percent A to fifty percent A between one and nine minutes, further increasing from fifty percent A to ninety-eight percent A between nine and twelve minutes, maintaining it at ninety-eight percent for twelve to thirteen point five minutes, and then slowly decreasing back to two percent between thirteen point five and seventeen minutes (Zelena et al*.* 2009).

The mass spectrometry analysis was conducted using a Thermo Orbitrap Exploris 120 mass spectrometry detector (Thermo Fisher Scientific, USA) with an electrospray ion source (ESI) operating in both positive and negative ion modes. The positive ion spray voltage was set at 3.50 kV, while the negative ion spray voltage was -2.50 kV. Gas pressures of 30 arbs and 10 arbs were maintained for sheath and auxiliary gases, respectively. The capillary temperature was set to 325℃. Data acquisition involved first-level full scanning at a resolution of 60,000 in the m/z range of 100 ~ 1000 using HCD for second-level fragmentation with a collision voltage of 30%. The second-level resolution was set at 15,000. The first four ions were fragmented before signal collection. Dynamic exclusion methodology, as described by Want et al. and Masson et al. in their 2013 publication, was implemented to enhance data analysis efficiency and reduce redundancy in MS/MS information (Want et al. 2013).

The original mass spectrometry file was converted to the mzXML file format using the MSConvert tool in the Proteowizard package (v3.0.8789). Peak detection, filtering, and alignment were performed using the RXCMS software package to generate a quantitative list of substances. Substances were identified by searching public databases such as HMDB, MassBank, LipidMaps, mzCloud, KEGG, and a custom material bank with parameters set at ppm < 30.

### Statistical analyses

The chemical composition and fermentation indexes were analyzed using IBM SPSS 23.0 software. A two-factor ANOVA was conducted to evaluate the effect of a 2 (silage additives) × 3 (silage days) factorial analysis. Duncan's multirange test was then used to assess the mean antioxidant mono-factor, with significance set at *P* < 0.05. Metabolites were analyzed using Orthogonal partial least squares discriminant analysis (OPLS-DA) with SIMCA. According to univariate statistical analysis, T-test is used to calculate P-value values, OPLS-DA dimensionality reduction method is used to calculate variable projection importance (VIP) and the impact strength and explanatory power of each metabolite component content on sample classification discrimination are measured to assist in the screening of biomarker metabolites. When the *P* < 0.05 and the VIP > 1, it is considered that the metabolite molecule has statistical significance.

## Results

### Characteristics of oat raw materials

The chemical compositions of oat silage material before ensiling are shown in Table 1. It showed that DM content was 34.29%, and the pH value of the raw material was 6.26, with soluble carbohydrate content and crude protein content at 5.87% DM and 6.99% DM, respectively. Additionally, the NDF and ADF contents were determined to be 43.70% DM and 23.69% DM, respectively. Furthermore, scavenging activities against DPPH and ABTS radicals were quantified as 0.80 μmol Trolox/g DM and 4.16 μmol Trolox/g DM.

**Table 1:** The chemical components of oat silage material before ensiling

Item	Bayou no.1
DM (% FW)	34.29 ± 1.20
pH	6.26 ± 0.10
WSC (%DM)	5.87 ± 1.90
CP (%DM)	6.99 ± 0.82
NDF (%DM)	43.70 ± 1.57
ADF (%DM)	23.69 ± 1.44

Data are means ± standard deviations of triplicate determinations

*DM* dry matter, *FW* fresh weight, *WSC* water-soluble carbohydrate, *EE* ether extract, *CP* crude protein, *NDF* neutral detergent fiber, *ADF* acid detergent fiber

### Fermentation characteristics of oat silage material during ensiling

The fermentation characteristics of oat silage are detailed in Table 2, where treatment, silage duration, and their interactions significantly impact levels of ammonia nitrogen and acetic acid (*P* < 0.05). Results reveal that the pH of the inoculated LAB (YiQing No.3) treatment group is lower compared to other groups, particularly the cellulase (M) group, showing a significant difference (*P* < 0.05). The concentration of NH_3_-N in the LAB treatment group is notably lower than in both the CK (control) group and M treatment groups. Even after 60 days of fermentation, the LAB and LM (YiQing No. 3 and cellulase) groups supplemented with lactic acid bacteria continue to exhibit significantly lower NH_3_-N content compared to other treatment groups during the same period (*P* < 0.05). In terms of organic acids, lactic acid content in the CK group is notably lower than that in the other three groups (*P* < 0.05), while acetic acid content remains higher across all treatment groups. As days progress, lactic acid content peaks at 30 d for all treatment groups before declining, while pH increases after 30 d. No significant differences are observed for butyric acid among treatments or over periods. Overall, additives improve the fermentation quality of oat silage. The pH value and lactic acid content are critical criteria for assessing the quality of silage fermentation. Specifically, high-quality silage should have a pH value lower than 4.2, and the amino acid content should constitute 3% to 8% of the DM. In conclusion, the additives significantly enhanced the fermentation quality of oat silage.

**Table 2:** The fermentation characters of oat silage after ensiling

Treatment	Ensiling day (d)	pH	NH_3_-N	Lactic acid	Acetic acid	Propionic acid	Butyric acid
			% DM				
CK^1^	7	3.76	1.75^ab^	5.19	0.75^b^	0.06	0.43
	14	3.68	0.54^ef^	5.89	0.55^bc^	0.03	0.04
	30	3.81	1.78^ab^	5.18	1.09^a^	0.15	0.05
	60	3.84	2.05^a^	4.52	1.10^a^	0.10	0.07
LAB^1^	7	3.66	0.49^f^	5.87	0.17^f^	0.05	0.04
	14	3.63	1.66^b^	6.71	0.20^f^	0.04	0.04
	30	3.80	0.59^def^	6.51	0.25^ef^	0.12	0.05
	60	3.84	0.78^def^	5.32	0.21^f^	0.12	0.09
M^1^	7	3.80	1.36^c^	5.47	0.24^ef^	0.06	0.05
	14	3.80	1.48^bc^	5.82	0.49^cd^	0.07	0.09
	30	3.82	1.22^c^	6.42	0.35^cdef^	0.05	0.07
	60	3.8	1.76^ab^	6.36	0.45^cde^	0.11	0.06
LM^1^	7	3.71	0.54^ef^	6.32	0.21^f^	0.04	0.04
	14	3.68	0.49^f^	6.18	0.24^ef^	0.18	0.04
	30	3.82	0.82^de^	7.02	0.32^def^	0.31	0.06
	60	3.87	0.87^d^	5.62	0.23^ef^	0.36	0.05
SEM		0.01	0.02	0.10	0.02	0.02	0.02
Treatment means^2^							
CK		3.77^ab^	1.53	5.20^b^	0.84	0.08^b^	0.15
LAB		3.73^b^	0.88	6.10^a^	0.21	0.08^b^	0.05
M		3.81^a^	1.46	6.01^a^	0.38	0.07^b^	0.07
LM		3.77^ab^	0.68	6.29^a^	0.25	0.22^a^	0.05
SEM		0.01	0.06	0.11	0.02	0.02	0.02
Day means^2^							
	7	3.73^b^	1.04	5.71^bc^	0.34	0.05	0.14
	14	3.70^b^	1.04	6.15^ab^	0.37	0.08	0.05
	30	3.81^a^	1.10	6.28^a^	0.50	0.16	0.06
	60	3.85^a^	1.36	5.46^c^	0.47	0.17	0.07
SEM		0.01	0.07	0.11	0.04	0.02	0.02
*P*-value							
T		0.015	< 0.001	0.001	< 0.001	0.011	0.393
D		< 0.001	< 0.001	0.012	0.006	0.055	0.507
T × D		0.304	< 0.001	0.166	0.002	0.622	0.370

Mean values with different letters in each column differ significantly (*P* < 0.05). T × D, the interaction between treatments and ensiling days

*DM* dry matter, *NH*_*3*_*-N* ammonia nitrogen, *SEM* Standard Error of Mea, *T* treatments, *D* ensiling days

^1^CK, control group. LAB, YiQing No.3 treatment group. M, Cellulase treatment group. LM, YiQing No.3 and cellulase synergistic treatment group. Data represent the means of 4 replicates (n = 4)

^2^Data represent the means of 16 replicates (n = 16)

The nutrient composition of oat silage is presented in Table 3. DM content was notably higher in the LAB and LM groups compared to the CK group (*P* < 0.05), with DM retention increasing as fermentation periods lengthened, potentially due to sampling errors. Both treatment and duration had significant effects on CP and WSC levels (*P* < 0.05). The CK group exhibited higher protein content than other groups, while the M group had the highest WSC content among all groups. EE and NDF were not influenced by silage duration or treatment (*P* > 0.05), whereas ADF was solely influenced by duration (*P* < 0.05), exhibiting a gradual decrease with longer fermentation days.

**Table 3:** The chemical composition of oat silage after ensiling

Treatment	Ensiling day (d)	DM %FW	CP	EE	NDF	ADF	WSC
			%DM				
CK^1^	7	33.74	7.97^bcd^	5.45	40.56	22.65	1.78^de^
	14	32.99	8.10^bc^	5.55	42.07	22.54	2.13^cde^
	30	33.38	8.36^b^	6.64	40.23	21.43	0.82^f^
	60	34.46	7.67^def^	5.74	41.47	21.62	1.59^ef^
LAB^1^	7	35.56	6.94^ghi^	4.82	41.66	22.53	3.28^ab^
	14	35.23	7.28^fg^	5.58	41.14	22.34	0.91^f^
	30	35.25	7.66^def^	5.06	39.94	21.12	2.54^bcd^
	60	35.74	7.19^g^	5.50	39.56	20.64	2.90^abc^
M^1^	7	34.30	6.64^i^	5.44	41.55	23.03	3.70^a^
	14	34.38	7.11^gh^	5.38	42.45	22.82	2.70^bc^
	30	34.37	7.60^def^	5.51	40.91	22.05	2.49^bcde^
	60	35.95	6.77^hi^	5.36	41.12	21.57	3.29^ab^
LM^1^	7	35.58	7.36^efg^	6.49	40.83	21.62	2.76^bc^
	14	35.31	7.86^cd^	5.79	42.48	22.65	2.29^cde^
	30	33.79	8.76^a^	5.99	39.67	20.93	2.48^bcde^
	60	37.95	7.72^cde^	5.60	40.41	19.88	2.47^bcde^
SEM		0.18	0.03	0.13	0.30	0.19	0.07
Treatment means^2^							
CK		33.64^b^	8.02	5.84	41.08	22.06	1.58
LAB		35.44^a^	7.29	5.24	40.58	21.66	2.41
M		34.75^ab^	7.03	5.42	41.51	22.37	3.04
LM		35.66^a^	7.92	5.97	40.85	21.27	2.50
SEM		0.20	0.06	0.12	0.28	0.19	0.10
Day means^2^							
	7	34.79^b^	7.25	5.55	41.12	22.45^ab^	2.88
	14	34.48^b^	7.58	5.78	42.04	22.58^a^	2.00
	30	34.20^b^	8.09	5.80	40.19	21.38^bc^	2.08
	60	36.03^a^	7.39	5.55	40.68	20.90^c^	2.57
SEM		0.21	0.07	0.13	0.27	0.18	0.11
*P*-value							
T		0.001	< 0.001	0.184	0.739	0.197	< 0.001
D		0.006	< 0.001	0.880	0.148	0.008	< 0.001
T × D		0.510	0.040	0.762	0.988	0.984	< 0.001

T × D, the interaction between treatments and ensiling days

*DM* dry matter, *FW* fresh weight, *CP* crude protein, *EE* ether extract, *NDF* neutral detergent fiber, *ADF* acid detergent fiber, *WSC* water-soluble carbohydrate, *SEM* Standard Error of Mean, *T* treatments, *D* ensiling days

^1^CK, control group. LAB, YiQing No.3 treatment group. M, Cellulase treatment group. LM, YiQing No.3 and cellulase synergistic treatment group. Data represent the means of 4 replicates (n = 4)

^2^Data represent the means of 16 replicates (n = 16)

### Antioxidant activity of oat silage

**Fig. 1:**
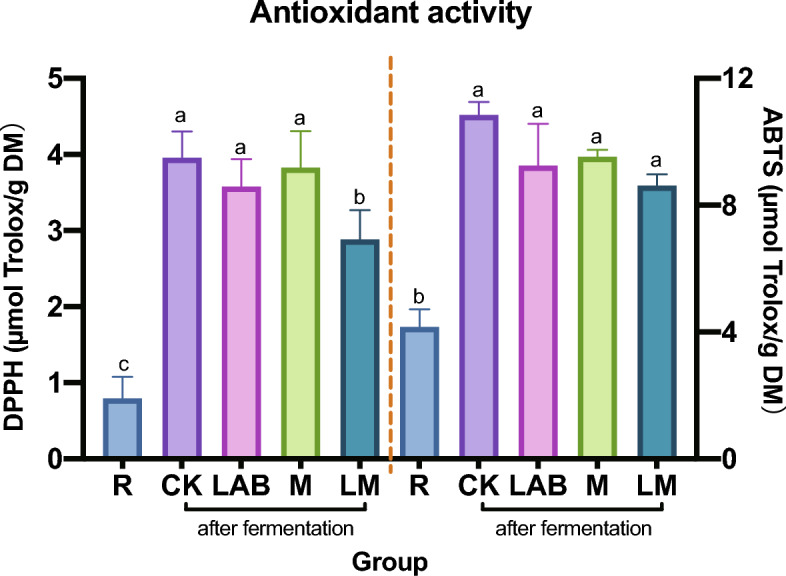
Antioxidant activity of different treatment groups. R, oat raw material. CK, control group. LAB, YiQing No.3 treatment group. M, Cellulase treatment group. LM, YiQing No.3 and cellulase synergistic treatment group. The color bars indicate identical processing conditions, while the lowercase letters denote the significance of the differences observed. On the left, the DPPH scavenging activity is presented, and on the right, the ABTS scavenging activity is illustrated

The antioxidant activity of oats before and after fermentation is depicted in Fig. 1, showing a notable increase in the antioxidant capacity of oat silage compared to the raw material (*P* < 0.05). This study evaluated antioxidant capacity using DPPH and ABTS radical scavenging activities. While there were no significant differences in DPPH radical scavenging ability between the CK group, LAB group, and M group, all three were notably higher than the LM group (*P* < 0.05). The ABTS radical scavenging ability did not vary significantly among the four groups post-fermentation (*P* > 0.05), but it was significantly higher than that of the raw materials (*P* < 0.05). After fermentation, the free radical scavenging ability of DPPH showed no significant differences among the CK group, LAB group, and M group; however, this ability was notably higher than that observed in the LM treatment group. In contrast, while the values of ABTS scavenging ability varied among the four treatments, no significant differences were observed.

### Changes in metabolites after oat silage

**Fig. 2:**
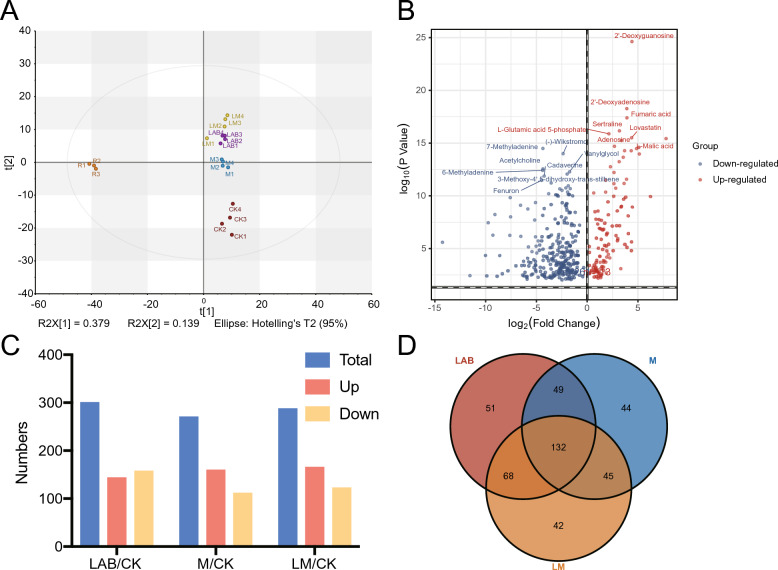
Overview of metabolomics analysis during ensiling process. (**A**) PCA score chart of oat raw materials and different treatments of oat silage samples. The orange dots represent the raw materials, the red dots represent the CK group, the purple dots represent the LAB group, the blue dots represent the M treatment group, and the yellow green dots represent the LM group (**B**) Volcano map of different metabolites between oat raw materials and oat silage samples with different treatments. The blue dots represent downregulated metabolites, red dots represent upregulated metabolites. Metabolites that show no significant differences are not displayed in the figure (**C**) Significant differences in the number of metabolites between each group and the CK group. Blue, red, and yellow represent the total amount, upregulated quantity, and downregulated quantity of differential metabolites between the two groups, respectively (**D**) Venn plot of the number of metabolites in three treatment groups. R, oat raw material. CK, control group. LAB, YiQing No.3 treatment group. M, Cellulase treatment group. LM, YiQing No.3 and cellulase synergistic treatment group

To investigate the factors influencing variations in antioxidant properties among different treatment groups, non-targeted metabolomics analysis of oat silage was conducted using LC–MS. Calculate the multiple of inter group differences using fold change (FC), |Log2 FC |≥ 1 can indicate upregulation or downregulation of metabolite content (Gao, Zhou et al. 2024). As shown in Fig. 2(A) and Fig. 2(B), the PCA score map revealed a distinct clustering of metabolites before and after the fermentation of oats, with a total of 822 metabolites detected. These metabolites were screened based on VIP > 1, *P* < 0.05, and |Log2 FC|≥ 1 criterion, leading to the identification of 374 significant differences in metabolites before and after fermentation, with 113 up-regulated and 261 down-regulated. The top ten up-regulated differential metabolites included carboxylic acids and derivatives, tetrahydronaphthalene, flavonoids, etc., ly: 2’-deoxyguanosine, 2’-deoxyadenosine, Fumaric acid, Sertraline, L-Glutamic acid 5-phosphate, Lovastatin, L-Malic acid, Adenosine, Malvidin 3-glucoside, Aucubin, The first ten down-regulated differential metabolites belonged to imidazole pyrimidines, organic nitrogen compounds, Flavonoids, etc., ly: 7-Methyladenine, (-)-Wikstromol, Acetylcholine, 6-Methyladenine, Vanylglycol, 3-Methoxy-4 ‘,5-dihydroxy-trans-stilbene, Cadaverine, Fenuron, Isovaleric acid, L-Phenylalanine. Compared with the CK group, the other three additive treatment groups also exhibited distinct clustering patterns. As shown in Fig. 2(C), in comparison with the CK group, the three treatment groups had respectively 300 differentially expressed metabolites (up 143; down 157), 270 species (up 159; down 111), and 287 species (up 165; down 122). As illustrated in Fig. 2(D), a total of 132 identical differential metabolites shared with the CK group were identified among the three treatments, and 51、44、42 unique types of metabolites could be found exclusively in each treatment, respectively. For detailed information on the types of differential metabolites, refer to supplement Table 1.

### Correlation between antioxidant activity and differential metabolites

**Fig. 3:**
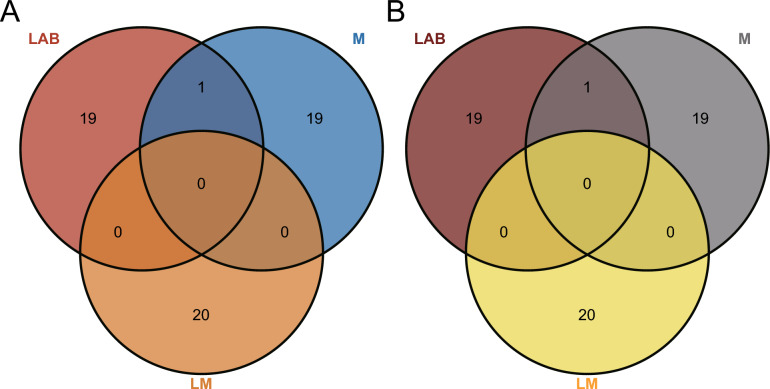
Overview of the Venn plot of the top 20 differentially expressed metabolites related to antioxidant activity in each treatment group with CK group (**A**)Venn plot of metabolites related to DPPH radical scavenging activity in different treatment groups with CK group (**B**) Venn plot of metabolites related to ABTS radical scavenging activity in different treatment groups with CK group. CK, control group. LAB, YiQing No.3 treatment group. M, Cellulase treatment group. LM, YiQing No.3 and cellulase synergistic treatment group

To investigate the impact of various additives on antioxidative metabolic pathways, a Pearson correlation analysis was performed on the identified metabolites in each group and their respective antioxidant activities. The top 20 metabolites in each group are presented in supplement Table 2 based on correlation analysis. Figure 3 illustrates the overlap of the top 20 unique metabolites associated with antioxidant properties across different treatments. Regarding DPPH and ABTS radical scavenging abilities were shown in Fig. 3A and B, only one common distinct metabolite was observed between the LAB group and M treatment group, while no shared distinct metabolite existed among other groups or treatments.

Supplement Table 2 presents the changes in the levels of the top 20 metabolites associated with antioxidant activity when compared to the CK group and the other three groups. In the CK group, the top 20 metabolites related to antioxidant activity primarily consisted of phenols, carboxylic acids and derivatives, flavonoids, etc. (refer to supplement Table 3 for detailed information). The differential metabolites associated with DPPH free radical scavenging activity in the LAB group included benzene and its substituted derivatives, flavonoids, organic oxygen compounds, etc. Compared to the CK group, there was a decreasing trend observed in the overall content of benzene and its substituted derivatives, while all flavonoid levels showed an increasing trend. Metabolites identified in both M group and LM group were mainly carboxylic acids and derivatives, as well as organic oxygen compounds. While there was an upward trend observed for these substances' contents in the M group, a downward trend was noted for them in the LM group. Regarding ABTS free radical scavenging activity, carboxylic acids, and their derivatives, along with flavonoids and organic oxides, were found to be predominant among metabolites detected in both LAB group and CK group; moreover, they exhibited an up-regulated pattern. Differentiated metabolites within Group M predominantly comprised carboxylic acids and derivatives, as well as organic nitrogen compounds; furthermore, there was a declining trend observed for these metabolite levels. Abundant differentiated metabolites were found within LM Group, including phenols, flavonoids, carboxylic acids and derivatives, fatty acids, and isoprene lipids. The contents of these substances demonstrated down-regulation effects.

## Discussion

Silage, a method that preserves nutrients in oats while enhancing their antioxidant activity, is widely recognized for its effectiveness in preserving fresh feed, and this method often modified with various additives to improve quality (Liu et al. 2021; Yi and Wang 2023). This study offers valuable insights into the application of oats in animal production.

In silage production, the initial DM value and WSC content are critical factors influencing silage fermentation. In this experiment, the DM value of the raw material was measured at 34.29 ± 1.20% FW. Natural fermentation was prone to butyric acid fermentation and nutrient loss. The WSC content was recorded at 5.87 ± 1.90% DM, which is slightly above the threshold for high-quality silage (Ni et al. 2017), thereby providing a substrate for microbial fermentation. As expected, the pH value of oat silage supplemented with lactic acid bacteria was found to be lower and significantly different from that of the M group. Following fermentation, the lactic acid content in the three additive treatment groups was significantly higher than that in the CK group. Additionally, up to 60 days of fermentation, the NH_3_-N level in the two treatment groups supplemented with lactic acid bacteria was significantly lower than that of the group without lactic acid bacteria, while the DM value was significantly higher than that of the CK group. Studies have demonstrated that the addition of lactic acid bacteria can enhance the DM preservation capacity of oat silage, improve lactic acid synthesis, and reduce pH levels (Xiong et al. 2022a, b). Xiong (2022) also noted that the NH_3_-N level decreased when *Lactiplantbacillus plantarum* was combined with cellulase, these findings suggest that the inclusion of lactic acid bacteria and cellulase in oat silage can improve nutrient preservation and decrease protein loss (Wang et al. 2019; Okoye et al. 2023). After 60 days of fermentation, the WSC content in the M group was significantly higher than that in the other groups during the same period, and some studies have indicated that the addition of cellulase significantly increases WSC content (Aniwaru et al. 1998). It should be noted that total mass data was not utilized in the calculation of the DM value in this experiment. Consequently, although the treatment group supplemented with lactic acid bacteria exhibited significant differences in DM value, the alterations in dry matter content following fermentation were primarily associated with water content and DM degradation. Therefore, this experiment did not furnish adequate evidence to support the assertion that lactic acid bacteria contribute to the reduction of nutrient loss. Nevertheless, it is important to acknowledge that all additive treatment groups in this study demonstrated positive effects on silage.

Following fermentation, it was observed that only the LM group exhibited significantly lower DPPH free radical scavenging activity compared to other groups treated with additives (*P* < 0.05), the value of other additive groups was lower than that of CK group but there was no statistically significant difference. Furthermore, the CK group demonstrated the highest levels of DPPH and ABTS free radical scavenging activities, while the LM group showed the lowest. Research indicates that microorganisms such as *Lactiplantbacillus plantarum* in the silage process break down large molecules into smaller ones and convert them into more useful forms (Ferreira-Lazarte, Plaza-Vinuesa et al. 2021, Sinaga, Tampubolon et al. 2022), leading to the degradation of antioxidants like polyphenols, vitamins, amino acids, and oligopeptides, thus reducing antioxidant properties (Cohen-Zinder et al. 2017). Some studies suggest that the abundance of microorganisms like *Lactiplantbacillus* increases notably after 60 days of fermentation (Huang et al. 2022), we speculated that microbial abundance and activity might explain why the antioxidant capacity of the additive group was lower than that of the CK group. however, it is important to note that no microbial experiments were conducted in this study. Additionally, the ABTS assay is suitable for both hydrophilic and lipophilic antioxidant systems, while the DPPH assay is more appropriate for hydrophobic systems due to the different free radical scavenging targets (Floegel et al. 2011), this distinction may account for the observed differences in results between the two antioxidant assays.

Untargeted metabolomic analysis was conducted to investigate the reasons behind the variations in antioxidant capacity among different additive treatment groups. The results revealed that the inoculation of various additives led to distinct regulatory effects on metabolite composition during the silage process. Guo and others observed 17 significantly different metabolites in whole-plant corn silage treated with different lactic acid bacteria (Guo et al. 2022). Su (2023) analyzed the metabolomic characteristics of whole corn silage inoculated with or without *Lactiplantbacillus plantarum*, showing a significant impact on metabolic components. In this study, Pearson correlation analysis revealed that the top 20 metabolites associated with antioxidant activity in each group exhibited distinct characteristics compared to the CK group. These findings imply that, although the differences in antioxidant activity may not be statistically significant, various additives influence the metabolic pathways of oat silage during fermentation, leading to the production of different metabolites linked to antioxidant strength in each group.

In terms of free radical scavenging activity of ABTS, carboxylic acids and derivatives represented the largest proportion among the top 20 metabolites associated with antioxidants across all treatment groups when compared to the CK group. Conversely, benzene and its substituted derivatives exhibited a significant impact on the scavenging activity of DPPH free radicals in the LAB group, while carboxylic acids and derivatives were predominant in the M and LM treatment groups. Alcohols and phenolic substances are known to produce carboxylic acids and derivatives through oxidation reactions (Du et al. 2023). Among the top 20 metabolites, the LAB treatment group exhibited an increasing trend in the relative contents of carboxylic acids and derivatives compared to the CK group, while phenolics and flavonoids also showed an overall increase. This suggests that fermentation could enhance the levels of phenolic and flavonoid metabolites, with lactic acid bacteria facilitating their conversion into carboxylic acids and derivatives, thus impacting antioxidant capacity. Benzene and substituted derivatives are generally involved in oxidation reactions (Du et al. 2023). The relative content of the LAB group decreased compared to the CK group under the influence of lactic acid bacteria, indicating a potential reduction in benzene and substituted derivatives during the silage process, thereby mitigating oxidation reactions (Min-Jie et al. 2016). Bao and others have suggested a significant positive correlation between the antioxidant activity of mulberry leaves and the total phenols and flavonoids present (Bao et al. 2016). He (2019) demonstrated that the supplementation of cellulase and *Lactiplantbacillus casei* can effectively enhance the flavonoid content and antioxidant activity of mulberry leaf silage. Furthermore, cooperative fermentation of bacterial enzymes was found to increase the extraction yield of total phenols from sage and rosmarinol by 30% (Weinberg et al. 1999). Zhang (2021) demonstrated that the use of *Lactiplantbacillus plantarum* 24–7 can elevate the levels of α-tocopherol, β-carotene, and polyunsaturated fatty acids in alfalfa silage, thereby enhancing overall antioxidant activity. The results indicate that various additives can influence the metabolic pathways during fermentation, thereby affecting the resulting metabolites and their antioxidant properties. Notably, fermentation has been shown to significantly enhance the antioxidant activity of oat silage compared to oat raw material. In this experiment, while the antioxidant activity across different treatment groups did not exhibit significant differences, the metabolic pathways underwent considerable changes. In the context of silage, metabolites associated with antioxidant activity are primarily concentrated in phenolic compounds and flavonoids. However, this study identified that the metabolites predominantly linked to antioxidant activity in oat silage are associated with carboxylic acids and derivatives, as well as benzene and substituted derivatives, as determined through non-targeted metabolomics analysis. Moving forward, we plan to develop highly specific detection methods utilizing targeted metabolomics technology to enhance sensitivity and accuracy in tracking changes in specific metabolites. Additionally, since the antioxidant activity among the treatment groups did not display significant differences, we intend to increase the number and concentration of treatments in future experiments and employ microbiome technology to connect the metabolic pathways more comprehensively with the resultant products.

## Conclusion

The addition of lactic acid bacteria and cellulase to oat silage can reduce protein loss, adding cellulase has been shown to increase the WSC content. In a word, both lactic acid bacteria and cellulase positively influence the fermentation quality of oat silage. Although no significant differences in antioxidant activity were observed among the various treatment groups in this experiment, notable changes in metabolic pathways were identified. Furthermore, two metabolites (carboxylic acids and derivatives and benzene and substituted derivatives) were identified through non-targeted metabolomics technology, both of which are strongly associated with the antioxidant activity of oat silage. In conclusion, the incorporation of lactic acid bacteria and cellulase as silage additives can significantly enhance both the fermentation quality and antioxidant capacity of oat  silage. This experiment provides a theoretical foundation for the precise and targeted regulation of oat silage additives to improve animal growth performance and resource utilization.

## Supplementary Information

Supplementary Material 1

## Data Availability

Not applicable.
