# Supplementary material for: Effects of lactic acid bacteria and cellulase additives on the fermentation quality, antioxidant activity, and metabolic profile of oat silage
Source: Bioresour Bioprocess. 2024 Oct 1;11(1):92. doi: 10.1186/s40643-024-00806-z (PMC11442794; doi:10.1186/s40643-024-00806-z)
Supplement: Supplementary file 1 — Supplementary Material 1 [file 40643_2024_806_MOESM1_ESM.pdf]

Supplemental 1. Top 50 differential metabolites compared with CK group

LAB	M	LM
Dihydrotestosterone	Dihydrotestosterone	Dihydrotestosterone
L-Theanine	L-Theanine	Kanosamine
Quinestrol	Dehypoxanthine futalosine	8-Amino-7-oxononanoate
Clofazimine	Clofazimine	Clofazimine
Dehydroepiandrosterone sulfate	Dehydroepiandrosterone sulfate	Dehydroepiandrosterone sulfate
cis-Aconitic acid	6-Acetamido-3-aminohexanoate	Nodakenin
S-Adenosylmethionine	cis-Aconitic acid	Prostaglandin J2
Timolol	Methoxamine	cis-Aconitic acid
2,4-Diketo-3-deoxy-L-fuconate	Timolol	S-Adenosylmethionine
3-Methyloxindole	Docosapentaenoic acid (22n-3)	Timolol
Docosahexaenoic acid	2,4-Diketo-3-deoxy-L-fuconate	2,4-Diketo-3-deoxy-L-fuconate
(+)-Demethoxyaschantin	3-Methyloxindole	3-Methyloxindole
Folic acid	Docosahexaenoic acid	Folic acid
Dodecanedioic acid	(+)-Demethoxyaschantin	3-(3,4-Dihydroxy-5-methoxy)-2-propenoic acid
Myristoleic acid	Folic acid	Cholesterol
Cholesterol	Myristoleic acid	Methotrimeprazine
Methotrimeprazine	Cholesterol	(R)-10-Hydroxystearate
Indican	Methotrimeprazine	Prostaglandin F3a
(R)-10-Hydroxystearate	D-Lysopine	Cycloheximide
Prostaglandin F3a	(R)-10-Hydroxystearate	Methyl hexadecanoic acid
Bepridil	Prostaglandin F3a	Sorbitol
Cycloheximide	Bepridil	Palmitic acid
4,4'-Dihydroxy-alpha-methylstilbene	Cycloheximide	Psoralidin
Exemestane	4,4'-Dihydroxy-alpha-methylstilbene	D-(+)-Glucose
Methyl hexadecanoic acid	Shikimic acid	p-Aminobenzoic acid
Alpha-Linolenic acid	N-Succinyl-L-citrulline	Lutein
Psoralidin	N2-gamma-Glutamylglutamine	Sphingosine 1-phosphate
D-(+)-Glucose	Methyl hexadecanoic acid	N(6)-[(Indol-3-yl)acetyl]-L-lysine
p-Aminobenzoic acid	Alpha-Linolenic acid	11Z-Eicosenoic acid
Strophanthidin	Palmitic acid	4'-Oxolividamine
11Z-Eicosenoic acid	Psoralidin	Metoclopramide
4'-Oxolividamine	D-(+)-Glucose	20-Hydroxyecdysone
3,4-Dihydroxymandelaldehyde	Strophanthidin	Phenyl acetate
Phenyl acetate	beta-D-Galactosyl-(1-_4)-L-rhamnose	Chrysoeriol
Chrysoeriol	11Z-Eicosenoic acid	Prostaglandin B2
Prostaglandin B2	Phenyl acetate	Guanidinosuccinic acid
Guanidinosuccinic acid	Prostaglandin B2	Imidazole-4-acetaldehyde
Imidazole-4-acetaldehyde	Guanidinosuccinic acid	4-Hydroxy-3-methoxy-cinnamoylglycine
Cornudentanone	(3S,4S)-3-Hydroxytetradecane-1,3,4-tricarboxyla	Asiatic acid
Phosphonoacetate	Cortol	Allantoin
Asiatic acid	12-Keto-tetrahydro-leukotriene B4	13S-hydroxyoctadecadienoic acid
Allantoin	Cornudentanone	Isovitexin
13S-hydroxyoctadecadienoic acid	Asiatic acid	trans-Cinnamate
Isovitexin	(S)-4-Hydroxymandelate	beta-D-Glucosamine
trans-Cinnamate	Allantoin	(2S)-Liquiritigenin
beta-D-Glucosamine	13S-hydroxyoctadecadienoic acid	Aldosterone
(2S)-Liquiritigenin	8-Isoprostane	gamma-Glutamylcysteine
gamma-Glutamylcysteine	beta-D-Glucosamine	L-Cystine
Tentoxin	(2S)-Liquiritigenin	Loganic acid

CK, control group. LAB, YiQing No.3 treatment group. M, Cellulase treatment group. LM, YiQing No.3 and cellulase synergistic treatment group.

Supplenmental 2. The changes in the levels of the top 20 metabolites associated with antioxidant activity compared to the CK group

	DPPH				ABTS			
	**Name**	**Class**	**Treatment[2)]**	**Type[3)]**	**Name**	**Class**	**Treatmen[2)]**	**Type[3)]**
	Dimethylbenzimidazole	Benzimidazoles	1	Down	Phosphoserine	Carboxylic acids and derivatives	1	Up
	Neocnidilide	Lactones	1	Down	9-Riburonosyladenine		1	Down
	Dodecanedioic acid	Fatty Acyls	1	Down	Diosmin	Flavonoids	1	Up
	Sorbitol 6-phosphate		1	Down	Antiarol		1	Up
	Secoisolariciresinol	Dibenzylbutane lignans	1	Up	Sucrose	Organooxygen compounds	1	Up
	Ketoleucine	Keto acids and derivatives	1	Down	L-4-Hydroxyphenylglycine		1	Up
	5-Carboxyvanillic acid	Benzene and substituted derivatives	1	Down	Clofazimine	Diazanaphthalenes	1	Down
	3,4-Dihydroxyphthalate	Benzene and substituted derivatives	1	Up	L-Histidine trimethylbetaine	Carboxylic acids and derivatives	1	Up
	Eupatilin	Flavonoids	1	Up	(Z)-4-Hydroxy-6-dodecenoic acid la	Lactones	1	Up
**LAB[1)]**	o-Toluate	Benzene and substituted derivatives	1	Down	Lactose 6-phosphate	Organic oxides	1	Up
	Acetylcholine	Organonitrogen compounds	1	Down	Epsilon-caprolactam	Lactams	1	Down
	Syringic acid	Benzene and substituted derivatives	1	Down	2-Aminophenol	Industrial Chemicals	1	Up
	Malvidin-3-glucoside	Flavonoids	1	Up	(E)-3-(4-Hydroxyphenyl)-2-propenal	Cinnamaldehydes	1	Up
	3-Hydroxyanthranilate	Benzene and substituted derivatives	1	Down	Sakuranetin	Flavonoids	1	Up
	2,4-Diketo-3-deoxy-L-fuconate		1	Down	Cantharidin		1	Up
	Bepridil	Benzene and substituted derivatives	1	Down	(+)-Bornyl diphosphate	Organic oxides	1	Up
	D-Galactose		1.2	Up	Cholesterol	Steroids and steroid derivatives	1	Down
	2'-Deoxyguanosine		1	Up	11-Dehydro-thromboxane B2	Fatty Acyls	1	Down
	Gluconic acid	Organooxygen compounds	1	Down	Vanylglycol	Phenols	1	Up
	Cytosine	Diazines	1	Up	Creatine	Carboxylic acids and derivatives	1.2	Down
	trans-Zeatin riboside	Azacyclic compounds	2	Up	Cycloheximide		2	Down
	L-Asparagine	Carboxylic acids and derivatives	2	Down	Acetylcholine	Organonitrogen compounds	2	Down
	D-Galactose		1.2	Up	S-Adenosylhomocysteine	5'-deoxyribonucleosides	2	Up
	(-)-Bornesitol	Organooxygen compounds	2	Up	N6-Acetyl-L-lysine	Carboxylic acids and derivatives	2	Down
	Histamine	Organonitrogen compounds	2	Down	Allantoin	Azoles	2	Down
	Asymmetric dimethylarginine	Carboxylic acids and derivatives	2	Up	Pyrophosphate	Non-metal oxoanionic compounds	2	Down
	Guanine	Imidazopyrimidines	2	Up	Methoxamine	Benzene and substituted derivatives	2	Down
	Isoquinoline	Isoquinolines and derivatives	2	Up	L-Valine	Carboxylic acids and derivatives	2	Down
	2-Keto-glutaramic acid	Keto acids and derivatives	2	Up	N-Acetyl-beta-alanine	Carboxylic acids and derivatives	2	Down
**M[1)]**	Phosphocreatine	Carboxylic acids and derivatives	2	Up	beta-D-Glucosamine	Organonitrogen compounds	2	Down
	N-Formyl-L-methionine	Carboxylic acids and derivatives	2	Up	N-Acetylaspartylglutamate	Carboxylic acids and derivatives	2	Up
	Mannitol 1-phosphate	Organooxygen compounds	2	Up	Timolol	Organonitrogen compounds	2	Down
	Shikimic acid	Organooxygen compounds	2	Down	Taurine	Organic sulfonic acids and derivative	2	Up
	Succinic acid	Carboxylic acids and derivatives	2	Up	Creatine	Carboxylic acids and derivatives	1.2	Down
	(R)-10-Hydroxystearate		2	Down	Caffeate		2	Up
	Gardenoside		2	Up	1H-Indole-3-acetamide	Indoles and derivatives	2	Down
	Orientin	Flavonoids	2	Up	Pantothenic acid	Alcohols and polyols	2	Down
	Cellobiose		2	Up	2-Hydroxy-3-(4-hydroxyphenyl)prop	Benzene and substituted derivatives	2	Up
	D-Glucose 1-phosphate	Organooxygen compounds	2	Up	6-Hydroxyhexanoic acid	Hydroxy acids and derivatives	2	Down
	Hydrocinnamic acid	Phenylpropanoic acids	2	Up	Methyl cinnamate		2	Up
	6-Phosphogluconic acid	Organooxygen compounds	3	Down	N(6)-[(Indol-3-yl)acetyl]-L-lysine	Azacyclic compounds	3	Down
	Pyroglutamic acid	Carboxylic acids and derivatives	3	Up	Neostigmine	Benzene and substituted derivatives	3	Down

	Asiatic acid		3	Down	Quercetin	Flavonoids	3	Down
	Isovitexin		3	Down	trans-Ferulic acid	Cinnamic acids and derivatives	3	Down
	Mannitol	Organooxygen compounds	3	Down	Tyrosol	Phenols	3	Down
	Quinoline	Quinolines and derivatives	3	Up	Lutein	Prenol lipids	3	Down
	Agmatine	Organonitrogen compounds	3	Up	Phytosphingosine	Organonitrogen compounds	3	Down
	L-Tyrosine	Carboxylic acids and derivatives	3	Down	N-Acetylglutamic acid	Carboxylic acids and derivatives	3	Down
	N-Acetyl-beta-alanine	Carboxylic acids and derivatives	3	Down	Dehydroepiandrosterone sulfate	Organic oxides	3	Down
	N6-Acetyl-L-lysine	Carboxylic acids and derivatives	3	Down	12-Hydroxydodecanoic acid	Hydroxy acids and derivatives	3	Down
**LM**[1)]	m-Cresol	Phenols	3	Up	Prostaglandin F3a	Fatty Acyls	3	Down
	Nodakenin		3	Down	Jasmonic acid	Fatty Acyls	3	Up
	N1-Acetylspermidine	Carboximidic acids and derivatives	3	Up	Loganic acid		3	Down
	Galactosylglycerol	Glycerolipids	3	Up	Kinetin	Imidazopyrimidines Flavonoids	3	Down
	1-Dehydro-[6]-gingerdione		3	Up	Baicalin	Carboxylic acids and derivatives	3	Down
	L-Cystine	Carboxylic acids and derivatives	3	Down	cis-Aconitic acid	Prenol lipids	3	Down
	Hordenine	Benzene and substituted derivatives	3	Up	Agnuside	Phenols	3	Down
	Arbutin	Organooxygen compounds	3	Up	Phenylephrine	Benzodioxoles Organonitrogen	3	Up
	Chrysoeriol	Flavonoids	3	Down	3,4-Methylenedioxyamphetamine	compounds	3	Down
	S-Adenosylmethionine		3	Down	Sphingosine		3	Down

1) LAB, YiQing No.3 treatment group. M, Cellulase treatment group. LM, YiQing No.3 and cellulase synergistic treatment group.
2) Number 1: different metabolites present in LAB group and CK group; Number 2: differential metabolites present in M group and CK group; Number 3: differential metabolites present in LM group and CK group
3)Type represents the change of content of each treatment group compared with CK group. Up, which means that the relative content of the treatment group is higher than that of the CK group. Down, indicating that the relative content of the treatment group is lower than that of the CK group.

Supplemental 3. Top 20 metabolites associated with antioxidant

ABTS[1]			DPPH[2]		
**Name**	**Class**	**Person[3]**	**Name**	**Class**	**Person[3]**
Carteolol	Azacyclic compounds	0.997994935	2'-Deoxyguanosine		0.99920812
Tryptophanamide	Azacyclic compounds	0.997038622	Hesperetin		0.998710971
N (omega)-Nitro-L-arginine methyl ester		0.993610132	Quinestrol	Steroids and steroid derivatives Indoles	0.996810026
Pyridoxamine 5'-phosphate Phenazine-1-	Pyridines and derivatives	0.992447612	N-Methyltryptamine	and derivatives Carboxylic acids and	0.99211757
carboxylate		0.989650908	Ornithine	derivatives Fatty Acyls	0.991926472
Dodecanoic acid	Fatty Acyls	0.987367283	9-OxoODE	Flavonoids	0.982398346
L-Glutamic gamma-semialdehyde	Carboxylic acids and derivatives	0.986814276	Isorhamnetin	Carboxylic acids and derivatives	0.980528094
5-Methylcytosine	Diazines	0.984272668	N-Acetylornithine	Organooxygen compounds	0.977640455
Tryptophanol	Indoles and derivatives Flavonoids	0.982123916	D-Glucose	Isoflavonoids	0.973690279
Epicatechin	Organic oxides	0.980506591	Daidzin	Phenols	0.970811051
6beta,11alpha-Dihydroxyprogesterone	Fatty Acyls	0.972720459	Moupinamide		0.967283329
Octadecanamide	Fatty Acyls	0.968550096	Yamogenin	Phenols	0.962839708
12,13-DHOME		0.967031377	Sinapyl alcohol	Phenols	0.961212868
2-Deoxystreptamine	Carboxylic acids and derivatives	0.966065906	3,4-Dihydroxyphenylacetate	Phenols	0.951415366
O-Acetylserine	Organooxygen compounds	0.965641743	Orciprenaline	Cinnamic acids and derivatives	0.947077886
(-)-Bornesitol	Flavonoids	0.958680055	trans-2-Hydroxycinnamate	Carboxylic acids and derivatives	0.946647624
Chrysoeriol	Organonitrogen compounds	0.956950188	Fumaric acid	Benzene and substituted derivatives	0.94547572
1,1-Dimethylbiguanide		0.956887187	Gentisic acid	Prenol lipids	0.945455349
16-Oxopalmitate	Keto acids and derivatives	0.956243069	all-trans-Retinoic acid	Fatty Acyls	0.943801472
Oxoadipic acid		0.949401092	Octadecanamide		0.939583959

ABTS, ABTS radical scavenging activity. DPPH, DPPH radical scavenging activity. Pearson, Pearson correlation coefficient.
